# Infusion of Sulfosuccinimidyl-4-[N-maleimidomethyl]cyclohexane-1-carboxylate-Conjugated MOG_35–55_-Coupled Spleen Cells Effectively Prevents and Reverses Experimental Autoimmune Encephalomyelitis in Mice

**DOI:** 10.1155/2015/129682

**Published:** 2015-07-14

**Authors:** Lanfang Zhang, Yixian Guo, Chang-Qing Xia

**Affiliations:** ^1^Department of Hematology, Xuanwu Hospital, Capital Medical University, Beijing 100053, China; ^2^Department of Pathology, Immunology and Laboratory Medicine, University of Florida College of Medicine, Gainesville, FL 32610, USA

## Abstract

In this study, we have evaluated our recently developed method for antigen-cell coupling using sulfosuccinimidyl-4-[N-maleimidomethyl]cyclohexane-1-carboxylate (sulfo-SMCC) heterobifunctional crosslinker in prevention and reversal of experimental autoimmune encephalomyelitis (EAE). We demonstrate that infusion of MOG_35–55_-coupled spleen cells (MOG-SP) significantly prevents and reverses EAE. Further studies show that the protected animals exhibit significantly delayed EAE upon EAE reinduction. Moreover, adoptive transfer of CD4+ T cells from the protected mice to naïve syngeneic mice renders the recipient mice resistant to EAE induction. Unexpectedly, CD4+ T cell proliferation is similar upon ex vivo stimulation by MOG_35–55_ amongst all groups. However, further analysis of those proliferating CD4+ T cells shows remarkable differences in Foxp3+ regulatory T cells (70% in MOG-SP groups versus 10–25% in control groups) and in IL-17+ cells (2-3% in MOG-SP groups versus 6–9% in control groups). In addition, we discover that MOG-SP treatment also significantly attenuates MOG_35–55_-responding IFN-*γ*-producing Th1 cells. These findings suggest that MOG-SP treatment induces EAE protective MOG_35–55_-specific regulatory T cells and suppresses EAE pathogenic Th17 and Th1 cells. Our study provides a novel approach for antigen-based EAE immunotherapy, which can potentially be translated into clinical application for immunotherapy of multiple sclerosis.

## 1. Introduction

Experimental autoimmune encephalomyelitis (EAE) is an induced autoimmune disease of the central nervous system (CNS) in rodent animals with the features of inflammation, demyelination, axonal loss, and gliosis [1]. EAE mouse model is one of the useful animal models for studying human multiple sclerosis (MS) because both conditions share common immunopathological processes [1, 2]. The most commonly used antigens to induce EAE mouse model are spinal cord homogenate (SCH), purified myelin, myelin protein such as myelin basic protein (MBP), proteolipid protein (PLP), and myelin oligodendrocyte glycoprotein (MOG), or the antigenic peptides from those proteins (e.g., MOG_35–55_) [3]. Immunologically, EAE and human MS result from the breakdown of self-tolerance and are thought to be associated with increased self-reactive Th17 cells [4–7], as well as impaired regulatory T cells [8, 9]. Thus, restoration of self-tolerance is a promising approach to the cure of EAE and MS.

EAE mouse model has been employed for testing preventive and therapeutic regimens [3, 10] including antigen-based immunotherapy [11–13]. Stephen Miller's group developed an effective approach in preventing and treating EAE by infusion of spleen cells coupled with myelin proteins treated by ethylene carbodiimide (ECDI) [14, 15]. This therapy is also effective in ameliorating other immune-mediated disorders such as type 1 diabetes [16, 17] and allograft rejection [18]. The results of a phase I clinical trial have shown that the treatment of ECDI-treated peripheral mononuclear cells coupled with a myelin peptide cocktail is well tolerated in MS patients, and a decrease in antigen-specific T cell responses is observed in patients receiving the highest cell doses [19]. The effect of immunotherapy using ECDI-treated spleen cells coupled with antigens has been considered to be associated with tolerogenic nature of apoptotic cells [20] because the process of ECDI-medicated antigen coupling leads to apoptosis of spleen cells [14]. Recently, we developed a novel antigen-cell coupling method with a much gentler heterobifunctional crosslinker, succinimidyl-4-[N-maleimidomethyl]cyclohexane-1-carboxylate (SMCC) or sulfosuccinimidyl-4-[N-maleimidomethyl]cyclohexane-1-carboxylate (sulfo-SMCC). We found that infusion of antigen-coupled spleen cells induced very potent antigen-specific T cell response (to be presented in another manuscript). The advantage of our antigen-coupling approach over ECDI-mediated antigen coupling is that the cells are not apoptotic upon antigen coupling. Thus, we can prepare antigen-coupled live cells or apoptotic cells depending on the treatment purposes. We are also able to compare antigen-coupled live and apoptotic cells in inducing antigen-specific immune responses.

In the current study, we seek to address whether treatment of SMCC-mediated myelin antigen-coupled spleen cells can lead to EAE protection. Our results showed that the treatment of ultraviolet-irradiated MOG_35–55_-coupled apoptotic spleen cells significantly prevented EAE or ameliorated ongoing EAE at early or established stage. Surprisingly, the treatment of MOG_35–55_-coupled live spleen cells provided EAE protection similar to that induced by antigen-coupled apoptotic spleen cells. Mechanistic studies showed that MOG_35–55_-coupled spleen cell treatment induced EAE protection may be associated with the induction of MOG_35–55_-specific Foxp3+ Tregs and the suppression of MOG_35–55_-specific Th17 and/or Th1 cells. Our study provides an excellent antigen-coupling method for immunotherapies of autoimmune diseases using antigen-coupled mononuclear white blood cells.

## 2. Materials and Methods

### 2.1. Mice

Female C57BL/6 mice at 6–8 weeks old were purchased from Charles River Animal facility in China (Beijing, China) and housed in the animal facility of Capital Medical University, Beijing. All mice were maintained under specific pathogen-free conditions and used following the Chinese governmental and Capital Medical University guidelines for animal welfare. This study was approved by the Capital Medical University Animal Ethics Committee.

### 2.2. Peptide and Reagents

The myelin oligodendrocyte glycoprotein peptide 35–55aa (MOG_35–55_, MEVGWYRSPFSRVVHLYRN GK) was synthesized by SBS Genetech (San Francisco, CA). The purity of these peptides was in the range of 95.18%. This is MHC-II presented peptide interacting with CD4+ T cells. Complete Freund's adjuvant (CFA) and pertussis toxin (PTX) were from Sigma-Aldrich. Reagents purchased from BD Bioscience (San Jose, CA) were* Mycobacterium tuberculosis* H37Ra, fixation/permeabilization kit, and leukocyte-activation cocktail (LAC). Sulfo-SMCC and Keyhole Limpet Hemocyanin (KLH) were from Thermo Scientific (Waltham, MA). The following fluorescent antibodies were used: CD4-PerCP (clone RM4-5, BD); IL-17-PE (clone TC11-18H10.1, Biolegend (San Diego, CA)); Foxp3-APC (clone 3G3, Miltenyi Biotec (San Diego, CA)). Foxp3/transcription factor staining buffer set used for Foxp3 intracellular staining was from eBioscience (San Diego, CA). Mouse CD4+ T cell enrichment kits (EasySep) were purchased from Stem Cell Biotech (Vancouver, Canada). Carboxyfluorescein Succinimidyl Ester (CFSE) used for cell tracking and T cell proliferation assay was from Life Technology (Grand Island, NY).

### 2.3. EAE Induction and Assessment

Female C57BL/6 mice were primed with an emulsion containing 1 mg/mL MOG_35–55_ and complete Freund's adjuvant (CFA) containing 5 mg/mL* Mycobacterium tuberculosis* H37Ra. A 200 *μ*L volume of emulsion was injected subcutaneously (s.c.) at three sites on the back of each mouse. Pertussis toxin (200 ng) in 200 *μ*L PBS was administered intraperitoneally (i.p.) on days 0 and 2.

Animals were observed daily for clinical signs of EAE and graded as follows: 0, no clinical sign; 1, partly limp tail; 2, totally limp tail; 3, partial hind limb paralysis or ataxia; 4, full paralysis of hind or forelimb affected; 5, moribundity or death.

### 2.4. Preparation of MOG-Coupled Spleen Cells

Spleens were removed from naïve female mice, and the RBCs were lysed using ACK lysis buffer (150 mM NH_4_Cl, 10 mM KHCO_3_, and 0.1 mM EDTA, pH 7.2–7.4). The splenocytes (10^8^) were incubated with sulfo-SMCC (0.05 mg/mL) and MOG_35–55_ (200 *μ*g/mL) for 1 h at room temperature. The MOG-coupled spleen cells (MOG-SPs) were washed thoroughly to eliminate possible contamination of soluble MOG and free sulfo-SMCC and the viability was around 90% by trypan blue staining. UVB-irradiated MOG-coupled spleen cells (UV-MOG-SPs) were prepared by ultraviolet B (UVB) irradiation (1200 mJ/cm^2^) following our protocol published previously [21]. The UVB-irradiated cells were placed on ice immediately after irradiation and injected into the mice within 2 h to avoid late stage apoptotic cells. The chemical reactions for MOG coupling to spleen cells with sulfo-SMCC were shown in supplemental Figure  1 in Supplementary Material available online at http://dx.doi.org/10.1155/2015/129682.

### 2.5. EAE Prevention and Reversal Experiments

#### 2.5.1. Prevention Experiment

C57BL/6 mice at 6 weeks of age were treated with intravenous injection of 1 × 10^7^ UV-MOG-SPs, MOG-SPs, spleen cells (SPs), or PBS once a week for two weeks. Then, all mice received EAE induction as described above. Thereafter, each group received two additional weekly corresponding treatments to consolidate the effect if any were induced during the first two weekly treatments. All mice were monitored daily and clinical manifestation (clinical scores) was recorded.

#### 2.5.2. Reversal Experiments

We performed EAE reversal experiments in mice at early and established stages. For early reversal experiment, we performed EAE induction as described elsewhere. Around one week before EAE onset, we administered intravenous injection of 1 × 10^7^/mouse MOG-SPs and UV-MOG-SPs, SPs, or PBS, for 5 times (days 5, 10, 15, 20, and 23). The EAE clinical presentation was monitored daily and recorded. For reversal of established EAE, we followed up a cohort of mice being subject to preinduction of EAE. Mice with clinical score 2 or above were randomly assigned to different groups as described above receiving the corresponding treatments twice a week for 3 weeks.

### 2.6. CD4+ T Cell Preparation and Adoptive Transfer

Spleen cells and lymph node from mice in prophylactic treatment group were collected 30 days after EAE induction. A single-cell suspension was prepared by mincing the organs in medium and filtered through a 70 *μ*m cell strainer. Erythrocytes were lysed with ACK lysis buffer. CD4+ T cells from different groups were magnetically purified using negative selection according to the manufacturer's instructions (EasySep kit from Stem Cell Biotech) with the purity > 95%. CD4+ T cells were intravenously injected into naïve C57BL/6 mice (5 × 10^6^/mouse), and EAE induction was performed on the same day using the protocol described above.

### 2.7. In Vitro Assessment of MOG Antigen-Specific CD4 T Cell Proliferation and Foxp3+ Regulatory T Cells

Mice from each group of preventive trail were sacrificed at the end of the experiment. Spleen cells were prepared as described above and then prelabeled with CFSE following the instruction from the manufacturer (Invitrogen). CFSE-labeled spleen cells (5 × 10^5^/well) were incubated with MOG_35–55_ (10 *μ*g/mL) or control antigen (KLH 10 *μ*g/mL) in a round-bottom 96-well culture plate. Triplicated wells were used for each condition. Four days later, the cultured cells were harvested, and part of the cells was subjected to flow cytometric analysis for CD4+ T cell proliferation (the dilution of CFSE on CFSE-labeled CD4+ T cells). The rest were stained for Foxp3 according to the instruction from the manufacturer (eBioscience, La Jolla, CA, USA) and analyzed by flow cytometry. CD4+ T cells were gated, and Foxp3 expression was analyzed for the proliferating and nonproliferating CD4+ T cells, respectively.

### 2.8. Flow Cytometric Analysis on Th1 and Th17 Cells

Mice from each group of preventive trail were sacrificed at the end of the experiment. Spleen cells were prepared and prelabeled with CFSE as described above. CFSE-labeled spleen cells (5 × 10^5^/well) were incubated with MOG_35–55_ (10 *μ*g/mL) or control antigen (KLH 10 *μ*g/mL) in a round-bottom 96-well culture plate. Triplicated wells were used for each condition. Four days later, leukocyte-activation cocktail (LAC) (1 *μ*L/mL) was added to the cultures for 4 additional hours. Thereafter, the cells were harvested and stained for CD4 using anti-CD4-PerCp (BD Bioscience) and then intracellular IFN-*γ* or IL-17 staining was performed using the protocol from the manufacturer (BD Bioscience). The IFN-*γ*+ CD4+ T (Th1) and IL-17+ CD4+ T (Th17) cells in MOG_35–55_-stimulated proliferating CD4+ T cells were analyzed by flow cytometry (FACS Canto, BD), which would represent MOG_35–55_ antigen-specific Th1 and Th17 cells, respectively.

### 2.9. Histology and Examination

For histological staining, mice were anesthetized and perfused with 0.9% sodium chloride solution followed by 4% paraformaldehyde. Lumbar regions of spinal cords were dissected and further fixed in 4% paraformaldehyde. Paraffin-embedded sections were stained with haematoxylin and eosin (H&E), Luxol fast blue (LFB), or neurofilament (NF) staining. We used H&E staining to examine the leucocyte infiltration and the diseased area showed loose tissue and vacuolization, inflammatory cell infiltration, or even formation of the perivascular cuffings. LFB staining was performed to assess the demyelination of spinal cord, and the blue dyeing became weak or even whitish in demyelination area. NF staining was used for examining axonal loss of the lesion site showing disappearance of coloring.

### 2.10. Statistical Analysis

Data were expressed as the mean ± SEM. Comparisons between groups were made by Student's* t*-test or one-way ANOVA for parameters with normal distribution and by Kruskal-Wallis test for parameters with nonnormal distribution. Significant difference was determined when *p* value was less than 0.05. Statistical analysis was performed using IBM SPSS Statistics 19.0.

## 3. Results

### 3.1. Administration of MOG_35–55_-Coupled Spleen Cells Significantly Prevents EAE

**Figure 1:**
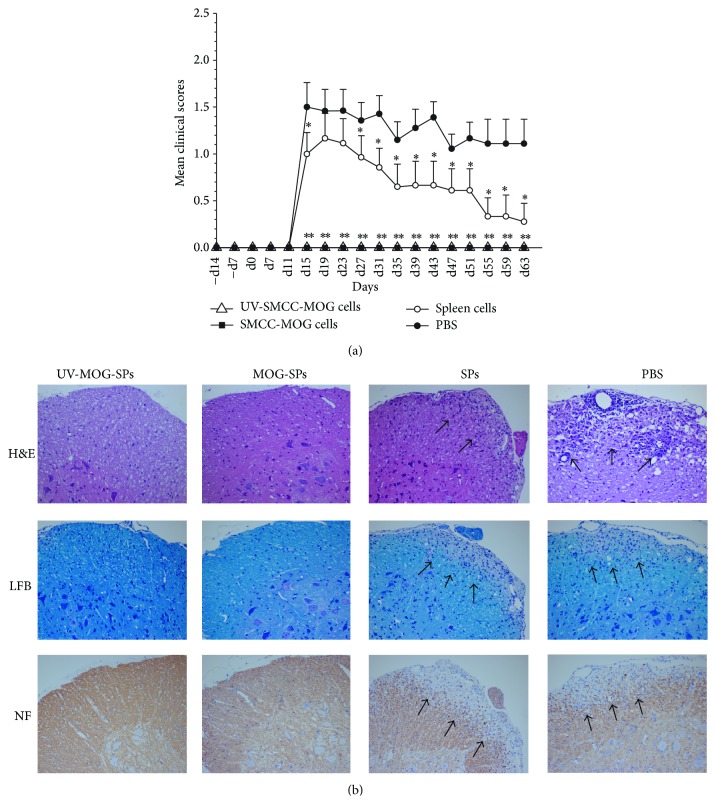
Effect of preadministration of MOG-coupled spleen cells on preventing EAE. (a) Female C57BL/6 mice were immunized with MOG_35–55_ peptide (200 *μ*g) emulsified in CFA containing* Mycobacterium tuberculosis* H37Ra on day 0. The mice received i.p. injection of 200 ng pertussis toxin on day 0 and day 2. Mice were randomly divided into four groups (10 mice/group) and given intravenous injection of 1 × 10^7^ different cells (UV-MOG-SPs, MOG-SPs, and SPs) or the same volume of PBS before EAE induction (designated as −d14 and −d7) and after EAE induction (days 0 and +7). All mice were monitored daily for over 2 months. The scores of treatment groups were compared with the scores of PBS treated group at each time point. ^*∗*^ *p* < 0.05; ^*∗∗*^ *p* < 0.01. (b) Histology examination of spinal cords from the mice receiving the treatments above was performed on day 30 (4 mice/group). H&E (upper panel), LFB (middle panel), and NF staining (bottom panel) were performed to examine mononuclear infiltration, demyelination, and axonal injury in the lumbar spinal cords. Original magnification ×20. The arrows showed a stronger infiltration, increased demyelination, and more axonal loss in SPs and PBS treated groups compared with MOG-coupled spleen cells treatment. Similar data were obtained from two additional independent experiments.

In this study, we tested our recently developed method using heterobifunctional protein coupling agent, sulfo-SMCC, to prepare MOG-coupled spleen cells for prevention of EAE. Given that apoptotic cells play an important role in inducing and maintaining immune tolerance [22], and SMCC-mediated protein coupling process did not cause cell death as described in Materials and Methods, we employed ultraviolet B (UVB) irradiation to induce apoptosis of MOG-SPs. To avoid injection of late stage apoptotic cells, we placed UVB-irradiated MOG-SPs on ice immediately after irradiation and injected the irradiated cells intravenously within 2 h to allow cell apoptotic process to start in vivo. As demonstrated in our previous study that majority of UVB-irradiated cells underwent apoptosis within 24 h [23], we found that if UVB-irradiated MOG-SPs were left in culture for 24 h, 90–95% of them became dead cells at early or late stages (data not shown). Four groups were included in this study: UV-MOG-SPs, MOG-SPs, SPs, and PBS. We treated female C57BL/6 mice with intravenous injection of spleen cells prepared as indicated above or PBS once a week for two weeks and then executed EAE induction by immunizing mice with MOG_35–55_ antigen as described in Materials and Methods. The day of EAE induction was defined as day 0. After EAE induction, to strengthen the induced preventative EAE effect, we administered two additional weekly treatments above, respectively. During two months of observation, we found that both MOG-SPs and UV-MOG-SPs completely prevented EAE with clinical scores of 0 (Figure 1(a)). Mice treated with SPs were also protected to some extent compared to PBS groups. Consistent with the clinical protection of EAE, spinal cord pathology of MOG-SPs and UV-MOG-SPs treated mice only showed mild infiltration of inflammatory cells and minor demyelination lesion, whereas PBS group exhibited significant inflammatory cell infiltration and demyelination damage (Figure 1(b)).

### 3.2. Administration of MOG_35–55_-Coupled Spleen Cells Leads to EAE Reversal at Early and Established Stages

**Figure 2:**
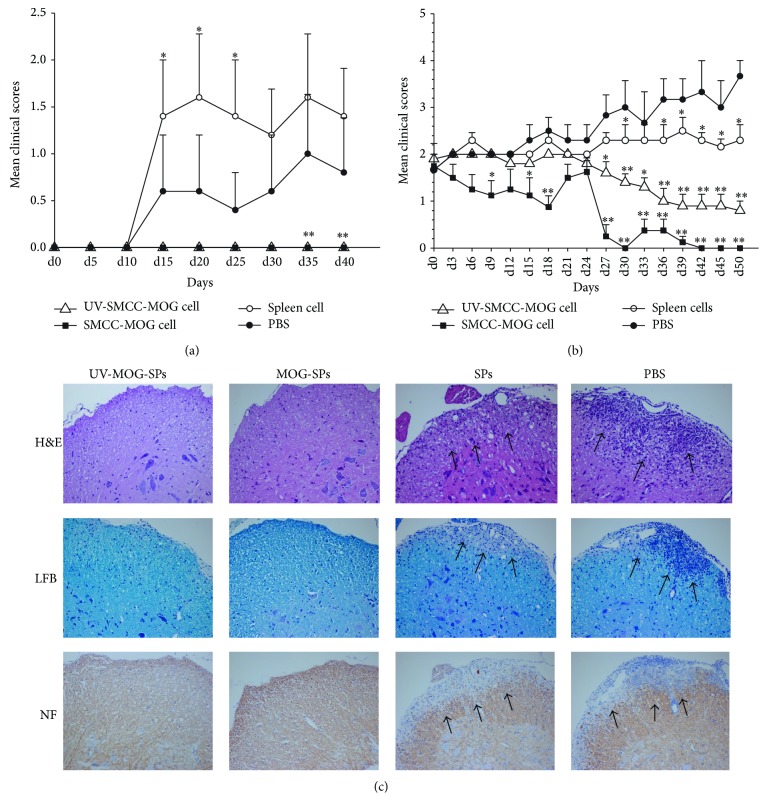
Infusion of MOG-coupled spleen cells reverses clinical EAE. (a) Naïve C57BL/6 mice were immunized as in Figure 1. Around one week before EAE onset, mice were randomly divided into four groups (UV-MOG-SPs, MOG-SPs, SPs, or PBS control, 5 mice/group) and given 1 × 10^7^ different cells or the same volume of PBS for 5 times (days 5, 10, 15, 20, and 23). The scores of treatment groups were compared to the scores of PBS treated group at each time point. ^*∗*^ *p* < 0.05; ^*∗∗*^ *p* < 0.01. (b) A cohort of mice was monitored after EAE induction. When the clinical score reached 2 or above, the EAE mice were randomly divided into four groups (5 mice/group): UV-MOG-SPs, MOG-SPs, SPs, or PBS. The mice in different groups received corresponding treatments twice a week for three weeks. The scores of treatment groups were compared with the scores of PBS treated group at each time point. ^*∗*^ *p* < 0.05; ^*∗∗*^ *p* < 0.01. (c) Sections from the spinal cords of EAE mice in late reversal experiments on day 50 (4 mice/group) were stained with H&E for inflammatory infiltrates (upper panel), LFB for demyelination (middle panel), and NF staining for axonal loss (bottom panel). Original magnification ×20. The arrows indicate areas of lesion with infiltrated cells, demyelination, and axonal loss. One representative animal of each group was exhibited. Similar results were obtained from two additional independent experiments.

The above results demonstrated that MOG_35–55_-coupled spleen cells completely prevented EAE, suggesting that SMCC-mediated MOG_35–55_-coupled spleen cell treatment was highly effective in EAE prevention. It is of great interest to determine whether MOG_35–55_-coupled spleen cell treatment is effective in reversing ongoing disease process of EAE. In this study, we assessed the effect of MOG_35–55_-coupled spleen cells on reversing EAE in both the early developing stage and established stage. For early EAE reversal, we performed EAE induction, but prior to overt clinical manifestation, we started to treat the mice with intravenous injection of UV-MOG-SPs, MOG-SPs, SPs, or PBS for 5 times. As shown in Figure 2(a), the administration of MOG-SPs or UV-MOG-SPs dramatically reversed early disease process, showing that none of the treated mice developed EAE during the observation period. The administration of SPs showed little effect compared to PBS groups. To further establish the efficacy of MOG_35–55_-coupled spleen cell treatment, we monitored a cohort of mice after EAE induction. Once clinical score reached 2 or above, we randomly assigned the EAE mice to different groups to receive intravenous injection of UV-MOG-SPs, MOG-SPs, SPs, or PBS, respectively, twice a week for 3 weeks. Strikingly, we found that EAE in both UV-MOG-SPs and MOG-SPs groups, the latter in particular, was significantly ameliorated (Figure 2(b)). EAE scores of SPs treated mice remained around 2 during the observation period, suggesting that spleen cells themselves might play a role in slowing down the disease process (Figure 2(b)). In contrast, EAE was worsened with time in PBS treated mice (Figure 2(b)). For the animals in the experiments of EAE reversal at late stage, we performed studies on spinal cord pathology to evaluate inflammatory cell infiltration and demyelination lesion. As shown in Figure 2(c), inflammation, demyelination, and axonal loss were hardly observed in the spinal cord white matter of UVB-MOG-SPs or MOG-SPs treated mice whereas pathological lesions were easily seen in SPs or PBS treated mice. The SPs treated mice still display substantial infiltration of inflammatory cells but with relative reduction compared to PBS treated mice. The severe demyelination was observed in both the spleen cell treated and PBS treated mice (Figure 2(c)). The above results indicate that administration of MOG_35–55_-coupled spleen cells can reverse EAE autoimmune process at both the early and late stages.

### 3.3. EAE Onset Is Significantly Delayed and Ameliorated in Protected Mice upon Rechallenge by MOG_35–55_ Immunization

**Figure 3:**
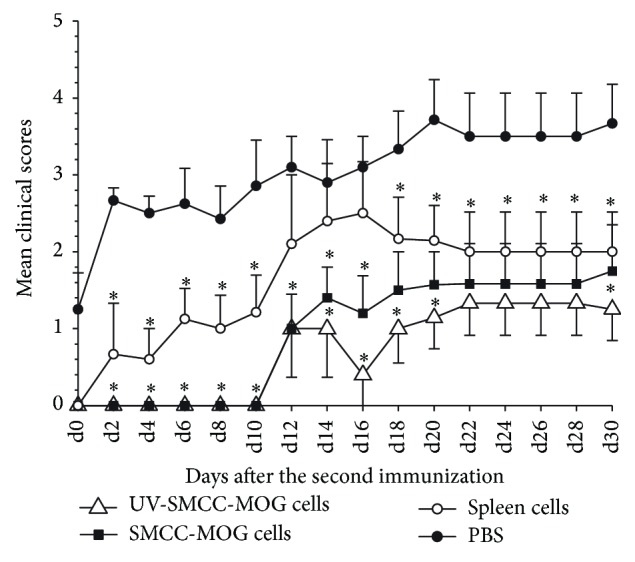
MOG-SPs treated mice develop significantly delayed and ameliorated EAE upon MOG challenge. Prevention experiments were performed as shown in Figure 1. EAE reinduction was performed 63 days after the first induction (10 mice/group). EAE development was monitored daily until day 30 after the second EAE induction. The scores of treatment groups were compared with the scores of PBS treated group at each time point. ^*∗*^ *p* < 0.05. Data shown were from a representative of three independent experiments.

To further determine the potency of EAE protection induced by MOG_35–55_-coupled spleen cells, we performed a prevention study as described above. Again, after about 2 months of observation following EAE induction, there was none showing clinical manifestations of EAE in both the MOG-SPs and UV-MOG-SPs treatment groups. However, overt EAE of various degrees was observed in the other two control groups. Thereafter, we performed an antigen challenge by EAE reinduction and monitored the changes of EAE development. We found that the mice having received the treatment of MOG-SPs or UV-MOG-SPs had significantly delayed and ameliorated disease upon EAE reinduction. In contrast, the MOG_35–55_ antigen challenge significantly worsened and accelerated the EAE in PBS treated mice. Compared to PBS treated mice, SPs treated mice also showed certain levels of resistance to EAE reinduction, which however was considerably weaker compared to MOG_35–55_-coupled spleen cell treated animals (Figure 3). UV-MOG-SPs treated animals appeared to have a greater resistance to EAE reinduction, but there was no significant difference as compared to MOG-SPs treated animals (Figure 3).

### 3.4. Transfer of CD4+ T Cells of MOG-SPs Treated Mice to Syngeneic Naïve Mice Leads to Significantly Delayed and Ameliorated EAE upon EAE Induction

**Figure 4:**
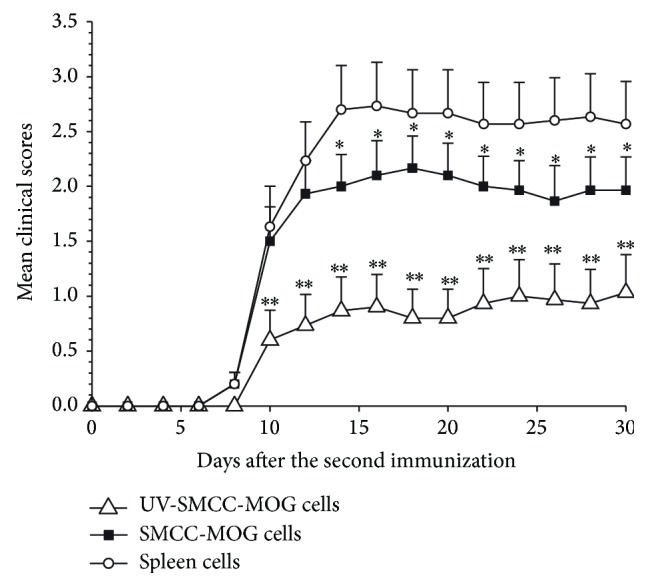
Adoptive transfer of CD4+ T cells leads to resistance to EAE induction. The splenic CD4+ T cells from three groups (treatment of SPs, UV-MOG-SPs, or MOG-SPs) were intravenously injected into naïve C57BL/6 mice (5 mice/group), respectively. After receiving CD4+ T cell transfer, all recipient mice underwent EAE induction on the same day. EAE development was monitored daily for 30 days. The scores of UV-MOG-SPs or MOG-SPs group were compared with the scores of SPs group at each time point. ^*∗*^ *p* < 0.05; ^*∗∗*^ *p* < 0.01. Similar data were obtained from two additional independent experiments, and the donor cells were prepared from different mice in these three experiments.

To confirm that MOG_35–55_-coupled spleen cell treatment induces MOG_35–55_ antigen-specific immunosuppressive regulatory T cells, we performed CD4+ T cell adoptive transfer study. In this study, we only employed one control group receiving adoptive transfer of CD4+ T cells from spleen cell treated mice as indicated. In accordance with the results shown in Figure 3, the adoptive transfer of CD4+ T cells prepared from UV-MOG-SPs or MOG-SPs treated animals significantly protected the recipient mice from developing EAE in contrast to transfer of CD4+ T cells from SPs treated animals (Figure 4).

### 3.5. MOG_35–55_ Antigen-Specific T Cells Respond to In Vitro MOG_35–55_ Stimulation with Similar Proliferation Rate amongst Different Groups

**Figure 5:**
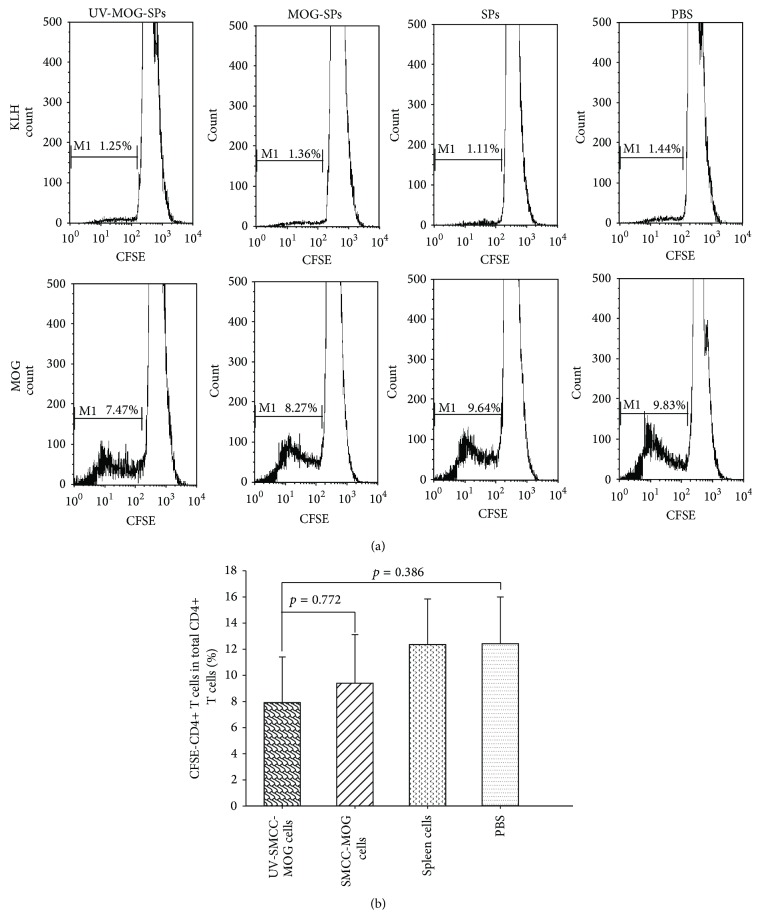
MOG-coupled spleen cells treatment induces similar MOG-specific CD4+ T cell proliferation in all groups. Spleen cells from each group in Figure 3 were labeled with CFSE; then CFSE-labeled spleen cells (5 × 10^5^/well) were stimulated with MOG_35–55_ (10 *μ*g/mL) or unrelated antigen KLH (10 *μ*g/mL) for 4 days. Thereafter, the cells were harvested and stained with anti-CD4-PerCp. CD4+ T cell proliferation (dilution of CFSE) was examined by flow cytometry by gating CD4+ T cells. (a) The flow cytometric data on proliferating CD4+ T cells/total CD4+ T cells (%) from one representative animal of each group were shown. (b) The data on proliferating CD4+ T cells with CFSE dilution in all CFSE-labeled CD4+ T cells from all animals (10 mice/group) were summarized. Statistical analysis results were depicted in the figures. Similar data were obtained from two additional independent experiments.

Based on the results demonstrated earlier in this study, it is speculative that the treatment of MOG-SPs or UV-MOG-SPs induces certain forms of antigen-specific immune tolerance. To address this question, we tested the capacity of spleen cells from different groups to respond to the stimulation of MOG_35–55_ in vitro. Spleen cells from all groups were prepared and labeled with CFSE and then stimulated with MOG_35–55_ or unrelated protein antigen, KLH, for 4 days. Thereafter, the cells were harvested and stained with fluorescent antibodies as indicated and CD4+ T cell proliferation was analyzed by assessing CFSE dilution using flow cytometry. To our surprise, we failed to observe any significant differences among all four groups in terms of CD4+ T cell proliferation (Figures 5(a) and 5(b)), although there was slight reduction of CD4+ T cell proliferation in UV-MOG-SPs treated group (Figure 5(a)). In addition, there was little, if any, CD4+ T cell proliferation upon KLH stimulation in all four groups (Figure 5(a)), suggesting that CD4+ T cell proliferation in response to MOG_35–55_ stimulation was antigen specific.

### 3.6. MOG_35–55_-Coupled Spleen Cell Treatment Induces MOG_35–55_ Antigen-Specific CD4+ Foxp3+ T Cells and Suppresses Pathogenic Th17 Cells

**Figure 6:**
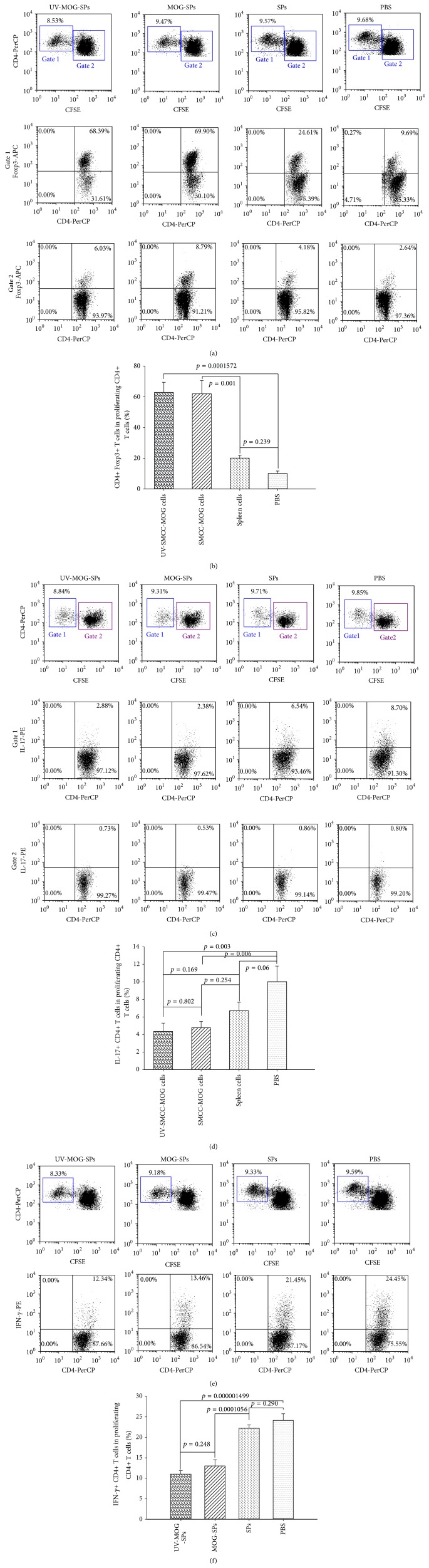
MOG-SPs treatment induces MOG-specific CD4+ T cell response with increased MOG-specific Foxp3+ Tregs and decreased MOG-specific Th17 cells and Th1 cells. (a) Spleen cells from each group were labeled with CFSE; then CFSE-labeled spleen cells (5 × 10^5^/well) were stimulated with MOG_35–55_ (10 *μ*g/mL) for 4 days. Then, the cells were harvested and stained with anti-CD4-PerCp and anti-Foxp3-APC following the protocol from the manufacturer (eBioscience). Foxp3+ CD4+ T cells in the proliferating and nonproliferating CD4+ T cells were analyzed by flow cytometry in gated CD4+ T cells (Gate 1 and Gate 2, resp.). (b) The summary of Foxp3+ CD4+ T cells in proliferating CD4+ T cells of each group (10 mice/group) and statistical analysis data were shown. (c) Spleen cells from each group were labeled with CFSE; then CFSE-labeled spleen cells (5 × 10^5^/well) were stimulated with MOG_35–55_ (10 *μ*g/mL) for 4 days. Then, leukocyte-activation cocktail (0.7 *μ*L/mL) (BD Bioscience) was added to the cultures for 4 h. Thereafter, the cells were harvested and stained for anti-CD4-PerCp and then stained intracellularly for IL-17 by anti-IL-17-PE. IL-17+ CD4+ T cells (Th17) in proliferating and nonproliferating CD4+ T cells were analyzed in gated CD4+ T cells (Gate 1 and Gate 2, resp.). (d) The summary of IL-17+ CD4+ T cells in proliferating CD4+ T cells of each group (10 mice/group) and statistical analysis data were depicted. (e) Spleen cells from each group were labeled with CFSE; then CFSE-labeled spleen cells (5 × 10^5^/well) were stimulated with MOG_35–55_ (10 *μ*g/mL) for 4 days. Then, leukocyte-activation cocktail (0.7 *μ*L/mL) (BD Bioscience) was added to the cultures for 4 h. Thereafter, the cells were harvested and stained for anti-CD4-PerCp and then stained intracellularly for IFN-*γ* by anti-IFN-*γ*-PE. IFN-*γ*+ CD4+ T cells in proliferating CD4+ T cells were analyzed in gated CD4+ T cells. (f) The summary of IFN-*γ*+ CD4+ T cells in proliferating CD4+ T cells of each group (10 mice/group) and statistical analysis data were depicted. This experiment was repeated twice with similar results.

The quantity of T cell proliferation appears to have no significant difference among different groups as shown in Figure 5. We were curious of the quality of CD4+ T cell proliferation in response to in vitro MOG_35–55_ stimulation. To address this issue, we evaluated the proliferating CD4+ T cells induced by stimulation of MOG_35–55_ in terms of Foxp3 expression. Surprisingly, majority of proliferating CD4+ T cells (around 70%) from UV-MOG-SPs or MOG-SPs were Foxp3 positive. The proliferating CD4+ T cells from SPs treated animals also displayed increased Foxp3+ cells (around 25%) in contrast to those from PBS treated group (Figures 6(a) Gate 1, and 6(b)), but with a degree significantly lower than UV-MOG-SPs or MOG-SPs treated group (Figure 6(a)). To rule out the global influence on Foxp3+ Tregs by the treatment, we also looked at the Foxp3 expression on nonproliferating CD4+ T cells. We found that the percentage of Foxp3+ cells was less than 10% for all groups (Figure 6(a), Gate 2), suggesting that MOG-SPs treatment induced MOG antigen-specific Tregs.

Th17 cells play a crucial role in the pathogenesis of EAE and MS [4, 24, 25]. To determine whether our treatment alters Th17 differentiation, we analyzed the proliferating CD4+ T cells stimulated by MOG_35–55_ using intracellular IL-17 staining assay. We found that IL-17+ proliferating CD4+ T cells (MOG_35–55_-specific Th17 cells) were significantly suppressed in animals receiving treatment of UV-MOG-SPs or MOG-SPs relative to those of PBS treated animals (*p* < 0.05, Figure 6(c), Gate 1). There was no significant difference between UV-MOG-SPs and MOG-SPs treated mice (Figure 6(d)). The percentage of IL-17+ proliferating CD4+ T cells in SPs treated group was comparable to that in PBS treated group (*p* > 0.05). It was also noted that there were very few IL-17-producing cells among the nonproliferating CD4+ T cells (Figure 6(c), Gate 2).

T helper 1 (Th1) cells secreting IFN-*γ* are thought to be another type of immunological players during EAE development [6, 26, 27]. We analyzed the proliferating CD4+ T cells stimulated by MOG_35–55_ using intracellular IFN-*γ* staining assay to determine whether our treatment alters IFN-*γ* level. As shown in Figures 6(e) and 6(f), spleen cells stimulated with MOG_35–55_ produced significantly higher levels of IFN-*γ*+ proliferating CD4+ T cells in SPs treated and PBS treated groups. On the contrary, IFN-*γ*+ proliferating CD4+ T cells were inhibited significantly from mice treated with UV-MOG-SPs or MOG-SPs. There was no significant difference between UV-MOG-SPs and MOG-SPs treated mice (Figure 6(f)). The percentage of IFN-*γ*+ proliferating CD4+ T cells in SPs treated group was comparable to that in PBS treated group (*p* > 0.05).

## 4. Discussion

Infusion of ECDI-treated myelin-coupled spleen cells has been demonstrated to effectively prevent EAE [14, 15, 20] or other immune-mediated disorders [16, 18], which is thought to be attributable to the antigen-coupled apoptotic cells induced in the process of antigen coupling [20]. Because of the harsh condition of ECDI-mediated antigen-coupling process, all antigen-coupled cells are dead. Therefore, it would be difficult to compare live and dead antigen-coupled cells in disease prevention and treatment using ECDI antigen-coupling approach. SMCC or sulfo-SMCC mediated antigen-cell coupling requires a mild condition that does not do any harm to the cells during antigen coupling [28]. This feature makes it possible to prepare antigen-coupled live and apoptotic cells. The latter can be readily achieved by UV irradiation [21, 23, 29, 30].

Because no cell death resulted from antigen coupling, SMCC-mediated antigen-cell coupling process was once used to prepare target cells in an in vitro assay for testing antigen-specific cytotoxic T cell-mediated cell killing [28]. However, there has been, thus far, no report using SMCC or sulfo-SMCC mediated antigen-coupled cells for induction of antigen-specific immune response or immune tolerance in vivo. In one paralleled study of ours, we successfully coupled exogenous model proteins such as ovalbumin (OVA) to mouse spleen cells, and upon intravenous injection of these OVA-coupled spleen cells to syngeneic mice, a potent OVA-specific immune response was induced (to be presented in another manuscript). These findings prompted us to test this approach in immune-mediated disease models. Given that infusion of ECDI-treated myelin-coupled spleen cells leads to EAE protection [14, 15, 31], we chose to use EAE model for the current study.

EAE is commonly employed as an animal model for human MS and has served as a powerful tool for studying disease pathogenesis as well as potential therapeutic interventions. In our current study, we showed that intravenous injection of UV-MOG-SPs not only prevented the onset of EAE in mice, but also effectively reversed or ameliorated the progression of ongoing EAE (Figures 1 and 2). Unexpectedly, infusion of live MOG-SPs also effectively induced EAE protection at a similar level to that induced by UV-MOG-SPs (Figures 1 and 2), suggesting that cell apoptosis is not necessary for antigen-coupled spleen cell-induced EAE protection, at least in this specific EAE mouse model. We further ruled out the effect induced by the infused spleen cells because injection of the same number of spleen cells not coupled with antigens only led to a minor protection (Figures 1 and 2). Although UV-irradiated spleen cells without coupling with MOG_35–55_ were not tested in this study, based on the notion that no difference was found between MOG-SP and UV-MOG-SP groups, treatment of UV-SP without coupling with MOG would behave similarly to the treatment of spleen cells. These results suggest that MOG_35–55_ antigens coupled to the infused spleen cells are crucial for inducing EAE protection. It is worth noting that in contrast to the previous report using multiple myelin antigens to prepare ECDI-treated antigen-coupled spleen cells [15] our approach is only utilizing one antigen, MOG_35–55_. The high EAE protective effect induced by the treatment of UV-MOG-SPs or MOG-SPs might be through antigen epitope spreading to influence other EAE pathogenic T cells [32–34]. As demonstrated in supplemental Figure 2 as well as in Stephan's study [35], the major splenocyte subsets express high levels of thiols. Because the vast majority of splenocytes are lymphocytes including T and B cells, it is speculative that MOG_35–55_-coupled T and B cells would be the major cell populations leading to EAE protection. However, this issue needs to be further addressed.

EAE and human MS have been demonstrated to be associated with the enhanced self-reactive Th17 and/or impaired Tregs [4, 8, 36]. The imbalance between Th17 and Tregs results in autoimmunity against CNS axonal antigens [9, 24]. Suppression of Th17 and augmentation of Tregs is an appealing strategy in managing EAE/MS [24]. To determine whether infusion of MOG-coupled spleen cells induced EAE protection is associated with increased Tregs and/or reduced Th17, we evaluated the CD4+ T cell response in vitro by the stimulation of MOG_35–55_ peptides presented by MHC-II in terms of T cell proliferation, development of Foxp3+ T cells and Th17 cells. To our surprise, CD4+ T cells proliferated to similar levels amongst all groups (Figure 5). These results suggest that EAE protection induced by the MOG_35–55_-coupled spleen cell treatment is not due to T cell anergy of MOG_35–55_-responding CD4+ T cells. To characterize the proliferating CD4+ T cells induced by MOG_35–55_ stimulation in vitro, we first looked at their Foxp3 expression. Intriguingly, Foxp3+ CD4+ T cells in proliferating CD4+ T cells were drastically enhanced in MOG_35–55_-coupled spleen cell treated mice in contrast to spleen cell treated and PBS treated mice (approximately 70% versus 25% versus 10%) (Figures 6(a) and 6(b)). Of interest, this change only occurred to MOG_35–55_-stimulated proliferating CD4+ T cells, because the percentages of Foxp3+ CD4+ T cells in nonproliferating CD4+ T cells were generally low (<10% (3–9%)) and equivalent for all groups (Figures 6(a) and 6(b)), which is close to the normal range (5–10%) in spleen cells [37]. These results indicate that MOG_35–55_-coupled spleen cell treatment has led to increased ratio of MOG_35–55_-specific Tregs in the total Tregs in vivo despite no difference in terms of the percentage of Tregs in CD4+ T cells amongst all groups (data not shown), and in vitro MOG_35–55_-stimulated T cell proliferation magnifies the positive rate of the antigen-specific T cells. It is also possible that precondition with MOG-SPs treatment confers a preferential differentiation of MOG_35–55_-responding CD4+ T cells to Tregs. Recent evidence shows that CD5 expression of CD4+ T cells makes the latter incline to Treg differentiation [38]. Further investigation is required to address this issue in our EAE protection model. Notably, MOG-SPs and UV-MOG-SPs induced similar levels of MOG_35–55_-specific Foxp3+ Tregs (Figures 6(a) and 6(b)), which could explain why both treatments protected EAE similarly. Additionally, it would be of great interest to learn whether MOG_35–55_-coupled spleen cell treatment suppressed MOG_35–55_-specific pathogenic Th17 cells and Th1 cells, which have also been thought to be associated with EAE immunopathogenesis [6, 26, 27]. Our results clearly demonstrate that IL-17+ CD4+ T cells and IFN-*γ*+ CD4+ T cells in MOG_35–55_-stimulated proliferating CD4+ T cells were significantly reduced compared to those in the control groups (SPs and PBS treated mice) (Figures 6(c)–6(f)). Very few IL-17+ CD4+ T cells were detected in nonproliferating CD4+ T cells, which show no difference amongst different groups (Figures 6(c) and 6(d)). These results suggest that the suppression of MOG_35–55_-specific Th17 and/or Th1 cells might have contributed to the EAE protection induced by the treatment of MOG-coupled spleen cells. Furthermore, certain resistance to EAE induction in the protected (Figure 3) or CD4+ T cell transferred (Figure 4) mice also strongly supports that MOG_35–55_-coupled spleen cell treatment induces EAE protective regulatory T cells. To further characterize MOG-SPs treatment induced Tregs, more accurate method, such as MOG_35–55_-tetramer, may be needed to assess whether there is increased rate of MOG_35–55_-specific Foxp3+ Tregs in total Tregs from MOG_35–55_-coupled spleen cell treated mice. Further studies will also be required to address why Tregs expansion by in vitro antigen stimulation only occurs in MOG_35–55_-coupled spleen cell treated mice and whether CD5 expression on CD4+ T cells of MOG-SPs treated mice is upregulated and instruct CD4+ T cells differentiating into Foxp3+ Tregs as demonstrated recently [38].

In the present study, we provide a simple and rapid antigen-coupling method for developing antigen-based immunotherapies for immune-mediated disorders, such as EAE. SMCC or its analogs mediated protein or peptide coupling with cells undergoes two “click-chemistry” reactions as depicted in Supplemental Figure 1. The ease, rapidity, simplicity, and relative irreversibility of “click” chemistry warrant the feasibility of this approach. Given that SMCC has been employed in clinic for drug delivery [39, 40], the approaches using SMCC-mediated antigen-coupled cells are highly clinically translatable for prevention and treatment of immune-mediated disorders.

## Supplementary Material

Supplemental Figure 1. The reaction scheme for conjugating MOG and spleen cells with Sulfo-SMCC. In the first reaction, a primary amine on a MOG's lysine resdues or N-terminus nucleophilically attacks SMCC, release sulfo-NHS, and thus forms a stable amide bond. In the second reaction, a cell surface sulfhydryl reacts with a maleimide functional group on the activated peptide, forming a viable protein-cell conjugate.
Supplemental Figure 2. Thiol (-SH) expression on different subsets of splenocytes and confirmation by 2-Mercaptoethanol neutralization. Freshly prepared splenocytes were incubated for 30 min with Fluorescein-5-maleimide, or Fluorescein-5-maleimide pre-incubated with 2-Mercaptoethanol (2-ME) in 1:2 molar ratio for 30 min. Then, the cells were stained with anti-CD4, CD8, B220, CD11b, CD11c and F4/80 fluorescent antibodies, respectively. The levels of mean fluorescent intensity (MFI) of Fluorescein-5-maleimide represent the levels of thiols on the cell surface. The results showed that the major populations of splenocytes expressed high-level thiols. Pre-incubation of Fluorescein-5-maleimide with 2-ME dramatically attenuated its binding to the cells, suggesting that Fluorescein-5-maleimide was truly reacting with the thiols on the cells surface.
