# Supplementary material for: Infusion of Sulfosuccinimidyl-4-[N-maleimidomethyl]cyclohexane-1-carboxylate-Conjugated MOG35–55-Coupled Spleen Cells Effectively Prevents and Reverses Experimental Autoimmune Encephalomyelitis in Mice
Source: J Immunol Res. 2015 Jul 14;2015:129682. doi: 10.1155/2015/129682 (PMC4516839; doi:10.1155/2015/129682)
Supplement: Supplementary file 1 — Supplemental Figure 1. The reaction scheme for conjugating MOG and spleen cells with Sulfo-SMCC. In the first reaction, a primary amine on a MOG's lysine resdues or N-terminus nucleophilically attacks SMCC, release sulfo-NHS, and thus forms a stable amide bond. In the second reaction, a cell surface sulfhydryl reacts with a maleimide functional group on the activated peptide, forming a viable protein-cell conjugate. Supplemental Figure 2. Thiol (-SH) expression on different subsets of splenocytes and confirmation by 2-Mercaptoethanol neutralization. Freshly prepared splenocytes were incubated for 30 min with Fluorescein-5-maleimide, or Fluorescein-5-maleimide pre-incubated with 2-Mercaptoethanol (2-ME) in 1:2 molar ratio for 30 min. Then, the cells were stained with anti-CD4, CD8, B220, CD11b, CD11c and F4/80 fluorescent antibodies, respectively. The levels of mean fluorescent intensity (MFI) of Fluorescein-5-maleimide represent the levels of thiols on the cell surface. The results showed that the major populations of splenocytes expressed high-level thiols. Pre-incubation of Fluorescein-5-maleimide with 2-ME dramatically attenuated its binding to the cells, suggesting that Fluorescein-5-maleimide was truly reacting with the thiols on the cells surface. [file 129682.f1.pdf]

Supplemental Fig 1

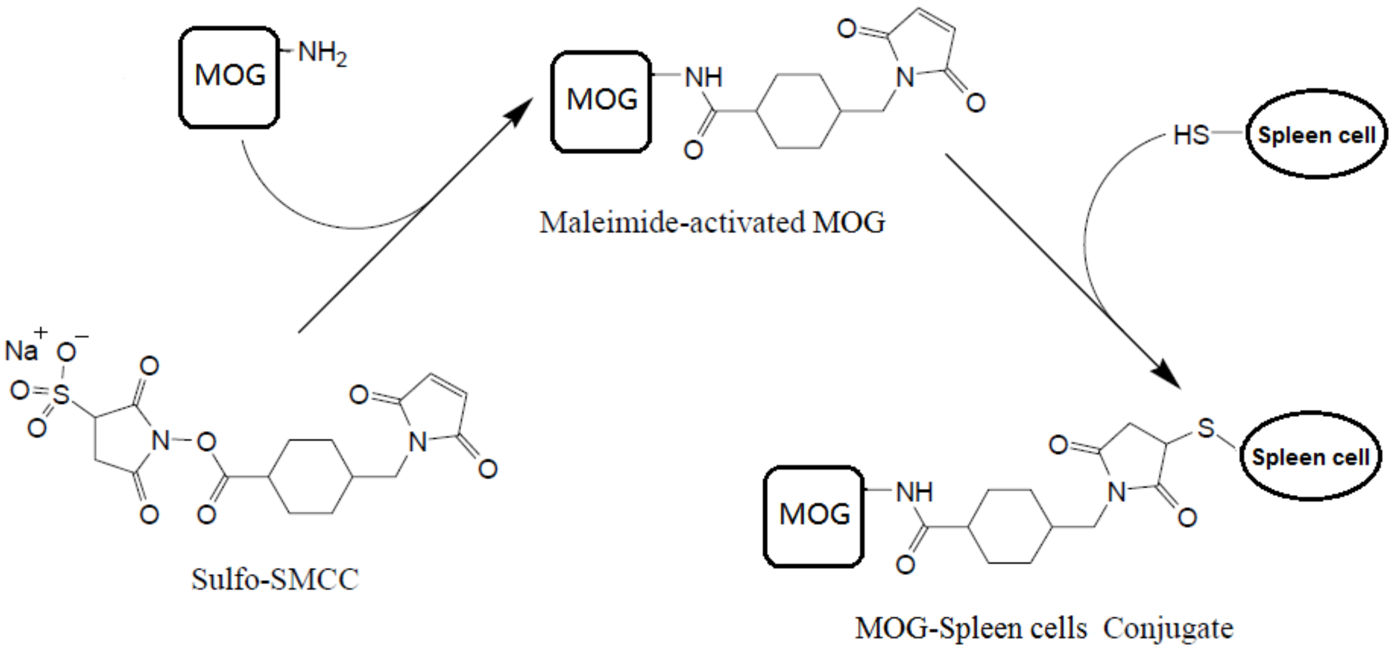

Supplemental Figure 1. The reaction scheme for conjugating MOG and spleen cells with Sulfo-SMCC. In the first reaction, a primary amine on a MOG's lysine resdues or N-terminus nucleophilically attacks SMCC, release sulfo-NHS, and thus forms a stable amide bond. In the second reaction, a cell surface sulfhydryl reacts with a maleimide functional group on the activated peptide, forming a viable protein-cell conjugate.

Supplemental Fig 2

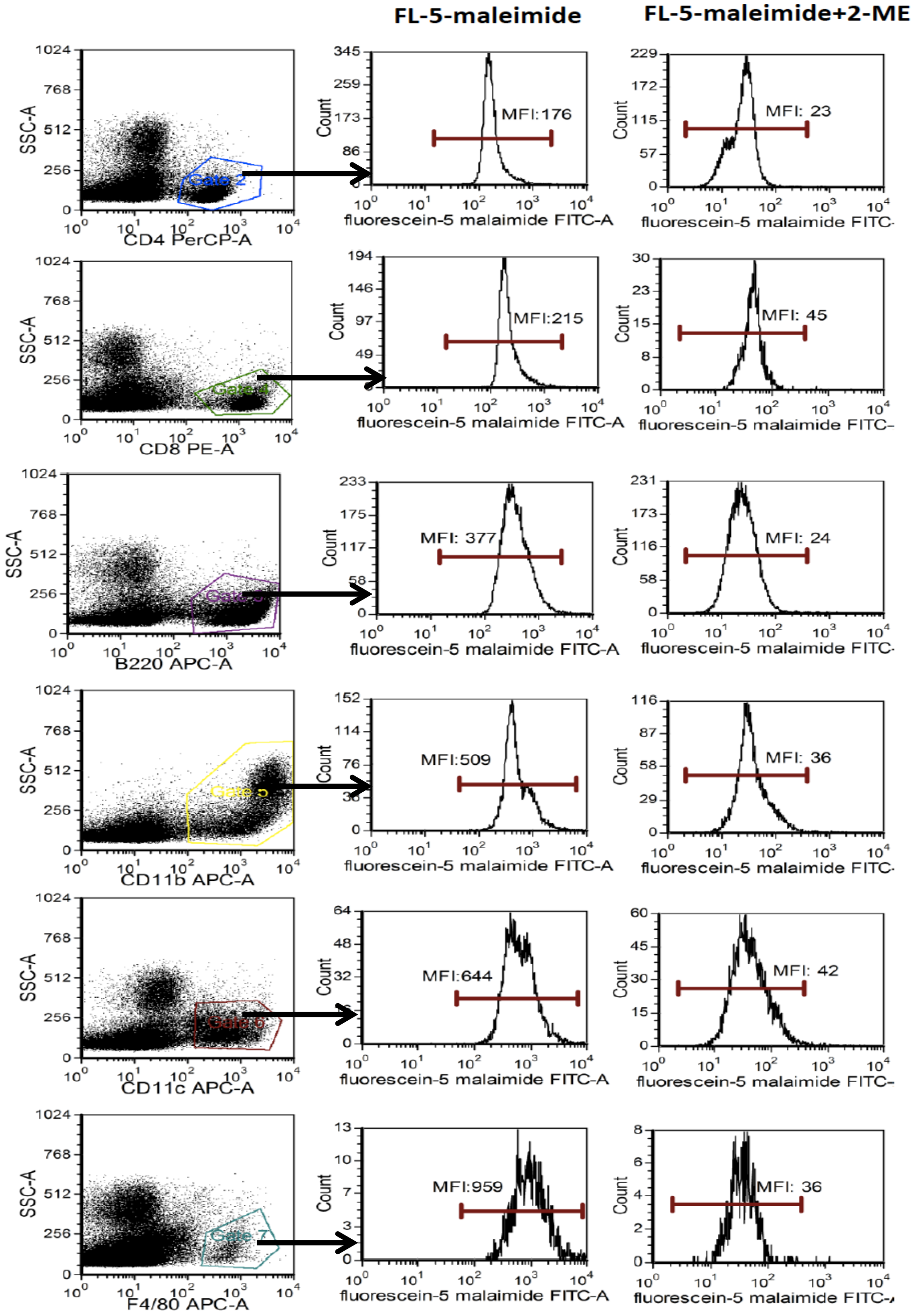

Supplemental Figure 2. Thiol (-SH) expression on different subsets of splenocytes and confirmation by 2-Mercaptoethanol neutralization. Freshly prepared splenocytes were incubated for 30 min with Fluorescein-5-maleimide, or Fluorescein-5-maleimide pre-incubated with 2-Mercaptoethanol (2-ME) in 1:2 molar ratio for 30 min. Then, the cells were stained with anti-CD4, CD8, B220, CD11b, CD11c and F4/80 fluorescent antibodies, respectively. The levels of mean fluorescent intensity (MFI) of Fluorescein-5-maleimide represent the levels of thiols on the cell surface. The results showed that the major populations of splenocytes expressed high-level thiols. Pre-incubation of Fluorescein-5-maleimide with 2-ME dramatically attenuated its binding to the cells, suggesting that Fluorescein-5-maleimide was truly reacting with the thiols on the cells surface.
